# Transcriptome Sequencing and Development of Genic SSR Markers of an Endangered Chinese Endemic Genus *Dipteronia* Oliver (*Aceraceae*)

**DOI:** 10.3390/molecules21030166

**Published:** 2016-02-23

**Authors:** Tao Zhou, Zhong-Hu Li, Guo-Qing Bai, Li Feng, Chen Chen, Yue Wei, Yong-Xia Chang, Gui-Fang Zhao

**Affiliations:** 1 Key Laboratory of Resource Biology and Biotechnology in Western China (Ministry of Education), College of Life Sciences, Northwest University, Xi’an 710069, China; woody196@163.com (T.Z.); lizhonghu@nwu.edu.cn (Z.-H.L.); bgq@ms.xab.ac.cn (G.-Q.B.); jjfl.007@163.com (L.F.); Estelleplus@163.com (C.C.); weiyue0101@sina.com (Y.W.); changyongxia_mia@sina.com (Y.-X.C.); 2 Xi’an Botanical Garden of Shaanxi Province, Xi’an 710061, China

**Keywords:** *Dipteronia*, transcriptome sequencing, *de novo* assembly, genic SSR, genetic diversity

## Abstract

*Dipteronia* Oliver (*Aceraceae*) is an endangered Chinese endemic genus consisting of two living species, *Dipteronia sinensis* and *Dipteronia dyeriana*. However, studies on the population genetics and evolutionary analyses of *Dipteronia* have been hindered by limited genomic resources and genetic markers. Here, the generation, *de novo* assembly and annotation of transcriptome datasets, and a large set of microsatellite or simple sequence repeat (SSR) markers derived from *Dipteronia* have been described. After Illumina pair-end sequencing, approximately 93.2 million reads were generated and assembled to yield a total of 99,358 unigenes. A majority of these unigenes (53%, 52,789) had at least one blast hit against the public protein databases. Further, 12,377 SSR loci were detected and 4179 primer pairs were designed for experimental validation. Of these 4179 primer pairs, 435 primer pairs were randomly selected to test polymorphism. Our results show that products from 132 primer pairs were polymorphic, in which 97 polymorphic SSR markers were further selected to analyze the genetic diversity of 10 natural populations of *Dipteronia*. The identification of SSR markers during our research will provide the much valuable data for population genetic analyses and evolutionary studies in *Dipteronia*.

## 1. Introduction

*Dipteronia* Oliver (*Aceraceae*) [1] is an endangered endemic genus found in southwestern and central China with only two living species, *D. sinensis* Oliver and *D. dyeriana* Henry [2]. Both species are members of the order Sapindales and family *Aceraceae* and are perennial woody plants with different natural ranges. In particular, *D. sinensis* is mainly found in central and southwestern China, whereas *D. dyeriana* is only located in the Yunnan Province in southwestern China. Fossil records indicate species of this genus also grew in North America during the Tertiary period [3]. Presently, the two living species have small population sizes due to deforestation and weak natural regeneration. *D. sinensis* and *D. dyeriana* are listed among Chinese Rare and Endangered Plants and China Species Red List with regard to being endangered, respectively [4].

Although *D. sinensis* and *D. dyeriana* live in different natural habitats, they still share some morphological similarities such as leaf shape and fruit character, although the mechanism of their divergent evolution remains unclear up to now. Therefore, to explore the genetic divergence between *D. sinensis* and *D. dyeriana* as well as to infer the genetic diversity of these species is still a valuable objective for us. Furthermore, it is also meaningful to trace the evolutionary dynamics of these two tertiary relict plants. Previous studies of *Dipteronia* have focused on universal genetic markers, morphologic divergence and/or seed physical and chemical properties [5,6,7,8] and only few genetic diversity studies were analyzed based on AFLP and cpSSR [9,10]. However, studies on speciation, demographic history, genomic variation and adaptive divergence of *Dipteronia* were not widely conducted, possibly due to limited genomic resources and genetic markers. So far only 116 nucleotide sequences from Sanger sequencing of *Dipteronia* have been deposited in the NCBI database and no ESTs were available yet. Hence, enriched genomic resources and genetic markers are much needed in order to decipher the genomic variation within the genus and investigate the genetic diversity in *Dipteronia*.

Due to their codominant and highly polymorphic nature, simple sequence repeats (SSRs) were commonly used for genetic diversity analyses and evolutionary studies [11,12,13,14,15,16,17,18,19]. Nevertheless, up to date, only a limited number of genomic SSRs were identified in *Dipteronia* according to the traditional FIASCO protocol [20,21]. No genic SSRs were developed yet. Therefore, it has become necessary to develop genic SSR markers for exploring genetic diversity and population structure of *Dipteronia* with neoteric technology.

Next-generation sequencing has greatly advanced our opportunities to obtain genome sequences and has also been widely used for various purposes in various species [22,23,24,25,26], but its application for large-scale population and phylogenetic studies is hindered by large genome sizes, DNA variation, polyploidy, and gene redundancy. Transcriptome sequencing has been an alternative to whole-genome sequencing because it can target genomic regions with corresponding EST sequences which can be exploited to develop EST-SSR markers. It is superior to conventional methods for developing SSR markers which were laborious and only yielded a few useful markers. Therefore, transcriptome sequencing has become a more effective and less expensive way to develop microsatellite markers. Research on non-model species have been investigated by using transcriptome sequencing and a number of microsatellite markers have been developed in these species [27,28,29,30,31,32,33,34]. In general, the genomic SSRs derived from traditional approaches were neutral anonymous markers which are not suitable for the assessment of functional diversity [35]. However, genic SSRs derived from the transcriptome sequences may actually regulate gene expression and function, making them a valuable resource for genetic studies and assessing functional diversity [36]. Furthermore, the genic SSR markers are transferable among distantly related species, whereas the genomic SSRs are not suitable for this purpose [37]. Overall, valid markers generated from transcriptome datasets will not only be important genomic resources but also be essential to explore evolutionary history and population genetics in plants, especially in endangered species.

To the best of our knowledge, to date no studies have reported the transcriptome sequencing of *Dipteronia*. The current study presents the transcriptomic profiling of material from the young leaves and fruits of two Chinese endangered endemic species of *Dipteronia* by using Illumina paired-end sequencing technology. Subsequently, a large set of genic SSR markers were developed for *Dipteronia* from transcriptome sequences. Overall, the transcriptomic resources and genic SSR markers collected herein would help us to guide further evolutionary and population genetic studies in *Dipteronia* and other relative species.

## 2. Results

### 2.1. De Novo Assembly of Illumina Sequencing Data

**Figure 1:**
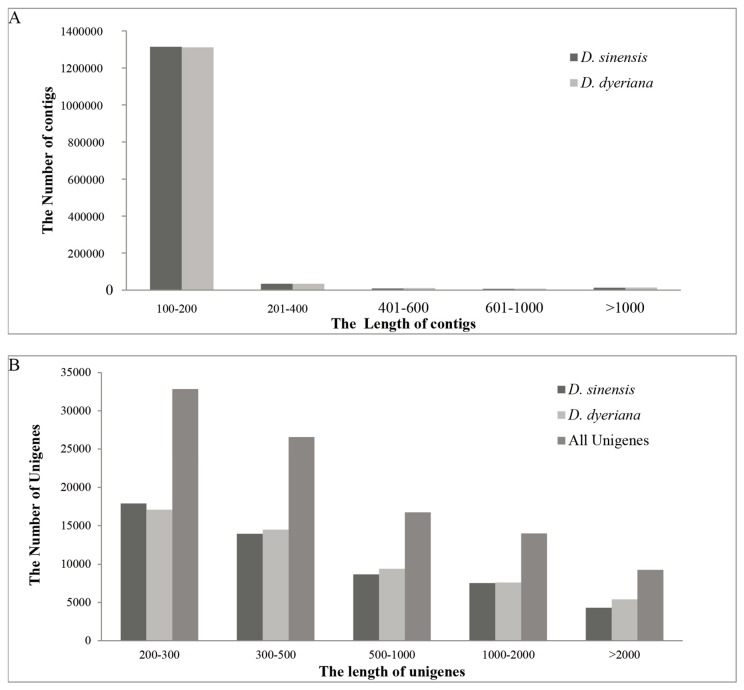
Length distribution of assembled contigs and unigenes. (**A**) Frequency distribution of the contig sizes from two *Dipteronia* species; (**B**) Size distribution of unigenes from two *Dipteronia* species.

After filtering and evaluating the raw reads, a total of 40.6 and 52.6 million reads were generated for *D. sinensis* and *D. dyeriana*, with 88.50% Q20 bases and 90.14% Q20 bases, respectively. The total length of the reads was approximately 9.4 gigabases (Gb, Table 1). The raw reads for *D. sinensis* and *D. dyeriana* were deposited in the NCBI Sequence Read Archive (SRA) under the accession numbers SRR2127986 and SRR2127991, respectively. Using the Trinity software, short read sequences from *D. sinensis* and *D. dyeriana* were assembled into 1,377,057 contigs and 1,378,610 contigs, respectively (Table 1). No or little difference was found in the contigs length of these two *Dipteronia* species (Figure 1). Using the paired end-joining, gap-filling and Trinity software, 52,351 and 53,983 unigenes were obtained from *D. sinensis* and *D. dyeriana*, respectively (Table 2). By further pooling and reassembling all the two individual pre-assembled unigene sets, 99,358 unigenes were recovered with a mean length of 783 bp and N50 value of 1452 bp (Table 2). The length distribution of unigenes for both species and combined non-redundant unigenes is consistent with the length distribution of contigs (Figure 1).

**Table 1:** Basic information of transcriptome reads, assembled contigs and unigenes for two *Dipteronia* species.

Species	Total Reads	Total Length of Reads	GC (%)	Q20 (%)	Number of Contigs	Total Length of Contigs
*D. sinensis*	40,615,432	4,101,851,805	45.50	88.50	1,377,057	114,648,897
*D. dyeriana*	53,620,610	5,314,269,235	45.21	90.14	1,378,610	117,846,656

**Table 2:** Summary of the unigenes from two *Dipteronia* species.

Unigenes Source	Total Number of Unigenes	Total Length of Unigenes	N50 of Unigenes	Mean Length of Unigenes
*D. sinensis*	52,351	39,234,224	1351	749
*D. dyeriana*	53,983	43,674,405	1519	809
All	99,358	77,820,010	1452	783

### 2.2. Functional Annotation for Non-Redundant Unigene Sequences

**Figure 2:**
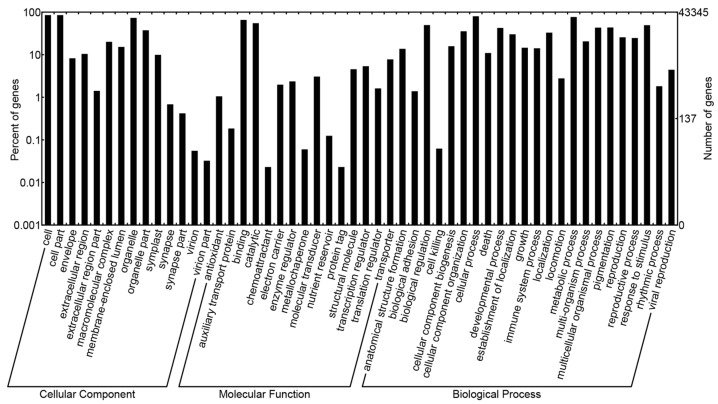
Gene Ontology (GO) classification of non-redundant unigene sequences from *Dipteronia.*

The sequence similarity searching for the non-redundant unigenes demonstrated that 52,789 unigene sequences had at least one blast hit against non-redundant (Nr), Cluster of Orthologous Group (COG), Swiss-Prot, Kyoto Encyclopedia of Genes and Genomes (KEGG), or Gene ontology (GO) database. Of these annotated unigene sequences, 22,106 unigenes with a length over 1000 bp. A total of 52,597 sequences were annotated in the Nr database and the three top-hit species for Nr annotation were *Theobroma cacao*, *Vitis vinfera*, and *Populus trichocarpa*. Based on COG functional classification, 18,218 sequences were assigned to 25 COG categories (Figure S1). Among these categories, the cluster for general function prediction was the largest group (4730, 25.96%); followed by translation, ribosomal structure and biogenesis (2185, 11.99%); replication, recombination and repair (2169, 11.91%); transcription (1946, 10.68%); posttranslational modification, protein turnover, chaperones (1706, 9.36%) and other classified groups. However, there were only five unigenes associated with nuclear structure but no unigenes were founded in the category of extracellular structures. For GO function, 43,345 unigene sequences were annotated based on sequence similarity. For the 14 level-2 categories in the cellular component category, cell part and cell were the most abundant terms; for the molecular function category, binding and catalytic activity were overrepresented among the 15 level-2 categories; for the biological process category, cellular process and metabolic process were the two most highly represented terms among the 23 level-2 categories (Figure 2). Using the Swiss-Prot database, 39,515 unigenes were annotated, and the minimum numbers of unigenes had blast hits against the KEGG database, since only 12,068 unigenes were annotated. With regard to the KEGG pathways analysis, the most representative were ribosome terms followed by glycolysis/gluconeogenesis and oxidative phosphorylation terms. Additionally, another KEGG pathway terms related to photosynthesis, respiration, terpenoid backbone biosynthesis, diterpenoid biosynthesis, anthocyanin biosynthesis and flavonoid biosynthesis were also recognized.

### 2.3. Detection of SSR Loci, Development and Validation of Microsatellite Markers

**Figure 3:**
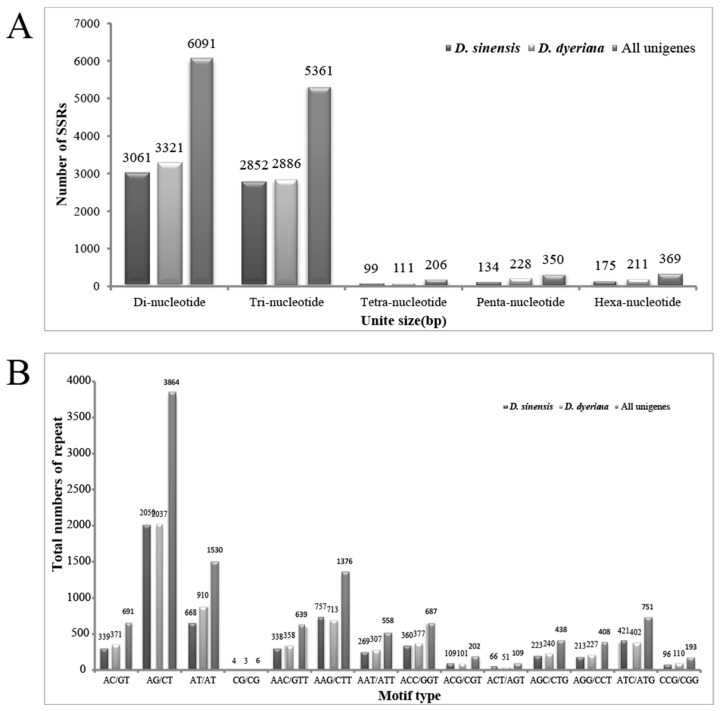
Frequency distribution of the SSRs identified in *Dipteronia* transcriptome. (**A**) Frequency distribution of the *Dipteronia* SSRs; (**B**) Total numbers of different SSR motifs in *Dipteronia*. Results of Di-nucleotides and Tri-nucleotides are represented.

In this study, a total of 6321/6757/12377 (*D. sinensis*/*D. dyeriana*/non-redundant unigenes) SSR loci were recognized using MISA perl script, of which 813/898/1658 unigene sequences contain more than one SSR. The average microsatellite density of the transcriptome in *Dipteronia* was one per 6207bp/7780bp/6287bp (Table 3). The most common type was di-nucleotide, followed by tri-nucleotide, hexa-nucleotide, penta-nucleotide and tetra-nucleotide (Figure 3). The number of repeat motifs per locus ranged from 5 to 12, in which SSRs with six repeats were the most abundant, followed by loci with five, seven and eight repeats. The number of SSR repeats of 12 was rare. The most abundant di-nucleotide repeat motif was AG/CT, followed by AT/AT, AC/GT; CG/GC was the least abundant. Among the tri-nucleotide repeat units, the dominant motif was AAG/CTT, followed by ATC/ATG and ACC/GGT (Figure 3).

**Table 3:** Summary of the microsatellite identified in the transcriptome.

Category	*D. Sinensis*	*D. Dyeriana*	All Unigenes
Total number of sequences examined	52,351	53,983	99,358
Total size of examined sequences (bp)	39,234,224	43,674,405	77,820,010
Total number of identified SSRs	6321	6757	12,377
Number of SSR containing sequences	5289	5614	10,253
Number of sequences containing more than 1 SSR	813	898	1658
Number of SSRs present in compound formation ^a^	29	27	717

^a^ The SSR locus contained at least two repeat motifs.

In order to validate microsatellite loci detected from non-redundant unigenes, a total of 4179 primer pairs were designed using Primer3 based on the 10,253 unigene sequences derived from the combined non-redundant unigene database. A total of 435 primers were randomly selected and validated in multiple individuals for both species. 374 primer pairs (86%) were successful in PCR amplification with genomic DNA from *D. sinensis* and *D. dyeriana*, whereas, the rest primer pairs failed to generate PCR products. Of the 374 primer pairs, 337 primer pairs amplified PCR products with the expected fragment size and the other 37 primer pairs produced fragments larger than the expected size. Afterward, all 337 SSR markers with the desired fragment size were screened in 44 individuals from both species to validate the polymorphism of the microsatellite markers. The results showed that 132 markers were polymorphic (Table S1). Of these 132 SSR markers, 91 SSR markers showed polymorphisms in *D. sinensis*, but monomorphism in *D. dyeriana*. Twenty-three SSR markers were polymorphic in *D. dyeriana* but monomorphic in *D. sinensis*. The remaining 18 loci showed high polymorphisms in both *D. sinensis* and *D. dyeriana* (Figure S2).

### 2.4. Genetic Diversity Analysis of D. sinensis and D. dyeriana

For evaluating the genetic diversity of *Dipteronia*, 44 individuals (Table 4) from 10 different natural populations of *Dipteronia* were collected and analyzed using 97 primer pairs selected from 132 polymorphic primer pairs. Based on 97 polymorphic primer pairs, the number of alleles (*Na*) per locus ranged from two to 13, with an average of 4.5 and the effective number of alleles per locus (*Ne*) varied from 1.05 to 8.60. The expected heterozygosity (*H_E_*) varied from 0.05 to 0.89 and observed heterozygosity (*H_O_*) ranged from 0 to 0.47. Shannon’s information index (I) values ranged from 0.11 to 2.24 with an average of 1.1.

**Table 4:** The information of 10 different populations used in genetic diversity analysis.

Population Code	Number of Individuals	Locations	Coordinates	Species
BX	4	Baoxing, Sichuan	30.54°N, 102.71°E	*D. sinensis*
CK	4	Chengkou, Chongqing	32.04°N, 108.72°E	*D. sinensis*
FY	4	Fuyan, Guizhou	27.34°N, 108.05°E	*D. sinensis*
FP	4	Foping, Shaanxi	33.68°N, 107.98°E	*D. sinensis*
WJG	4	Wanjiagou, Hubei	31.40°N, 110.55°E	*D. sinensis*
XE	4	Xuan‘en, Hubei	30.04°N, 109.67°E	*D. sinensis*
MZ	5	Mengzi, Yunan	23.40°N, 103.78°E	*D. dyeriana*
CJZ	5	Chenjiazha, Yunan	23.35°N, 103.97°E	*D. dyeriana*
ZCZ	5	Zhongcaozi, Yunan	23.27°N, 103.45°E	*D. dyeriana*
PB	5	Pingbian, Yunan	23.29°N, 103.88°E	*D. dyeriana*

**Figure 4:**
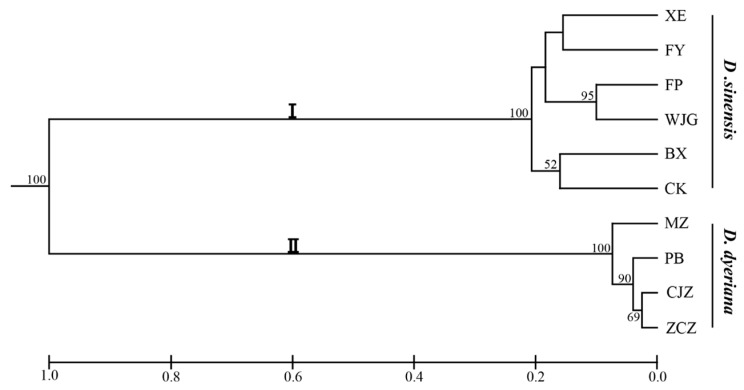
Dendrogram for 10 populations of *Dipteronia* from different areas based on 97 SSR loci. Bootstrap values (>50) were labeled on the branches from 10,000 re-samplings.

Polymorphism information content (PIC) values ranged from 0.04 to 0.87. The probabilities of deviation from Hardy-Weinberg equilibrium (HWE) proved that most of SSR markers did not significantly violate HWE (Table S2). A UPGMA dendrogram based on Nei’s genetic distance showed that all the populations were divided into two clusters, cluster I for *D. sinensis* and cluster II for *D. dyeriana* (Figure 4). Genetic diversity analyses were also separately implemented in each species based on these 97 SSR loci and the results indicated that 76 SSR loci showed a higher polymorphism in *D. sinensis* but only 14 SSR markers showed a higher polymorphism in *D. dyeriana* (Tables S3 and S4).

## 3. Discussion

### 3.1. Sequences Assembly and Annotation of Dipteronia

In the current study, similar numbers of unigene sequences were generated for both species after assembly, which was partly due to the fact the same tissues of both species were collected for sequencing. In parallel, the length distribution of contigs and unigenes in these two species was also coincidental, which indicated that the Illumina-based sequencing technology was successful and the assembly was relatively reliable. Therefore, the large number of unigenes obtained from this work could substantially increase the nearly non-existing genomic information of *Dipteronia*. In our sequence annotation attempt, more than half of the unigene sequences showed significant homology to genes in the Nr database. The relative high percentage of hits was partially caused by the increased number of long sequences in our unigene database (783 bp on average). In addition, our results indicated that 90.4% of unigenes over 1000 bp in length showed homologous matches in the Nr database. This is consistent with previous studies that reported longer unigene sequences were more likely to have BLAST matches in the protein databases [38,39]. The rest of the unigenes could not be functionally annotated due to no blast hit against database or they were matched to unknown proteins. This is not unexpected since there is no available genomic and transcriptomic information for *Dipteronia* or a comprehensive genomic resource for *Aceraceae*, therefore, many genes of *Dipteronia* were not accessible. Many unigenes could be assigned to a wide range of GO and COG classification, which manifested transcriptome data for this study including diversified transcripts. Numerous subcategories related to metabolism were abundant in the KEGG pathway since leaves are the most important metabolic organ for chlorophytes, several pathways associated with photosynthesis and respiration were recognized. Strikingly, pathways associated with terpenoid backbone biosynthesis and diterpenoid biosynthesis were also identified in present study. Previous phytochemical studies of *Dipteronia* revealed that terpenoids could be isolated from fruits and branches [40,41]. Therefore, some of the transcripts identified in our study might be involved in the synthesis of these phytochemicals. Additional KEGG pathways like anthocyanin biosynthesis and flavonoid biosynthesis were also highlighted in our analyses, suggesting that the identified transcripts might be involved in these biosynthesis.

### 3.2. Genic SSR Distribution and Frequency in Dipteronia Transcriptome

Many studies have demonstrated that transcriptome sequencing was a powerful tool for identifying SSR markers. Various SSRs derived from transcriptome sequencing have been extensively used in plant genetic diversity analyses [42,43,44]. However, until now no genic SSRs were identified and used to evaluate genetic diversity for *Dipteronia*. In this study, a lot of unigene sequences that contained microsatellite loci were detected. The percentage of SSRs contained sequences was higher than pigeon pea, *Cucurbita pepo* and celery [44,45,46], but was similar to chickpea [27]. The discrepant SSRs frequency might be mainly caused by the parameters of tools for searching microsatellite loci. In this work, mononucleotide repeats were excluded for detecting SSRs since they could be caused by base mismatch or sequencing errors and they were difficult to distinguish from polyadenylation products. The mean density of SSR distribution was one microsatellite locus per 6.2 kb/7.8 kb/6.3 kb. This density was less than the density in coffee (1/2.16 kb), and *Amorphophallus* (1/3.63 kb) [30,47], but it was higher than chickpea (1/8.66 kb) and *Arabidopsis* (1/14 kb) [48,49]. The different distribution frequency of microsatellite loci may be partially related to genome composition, data size, and microsatellite screening criteria. Dinucleotide repeats were the most abundant repeat type in our study. This finding was in accordance with previous studies using sesame, pigeon pea and poplar [44,50,51,52] but inconsistent with other plant species such as cabbage, peanut and *Brachiaria ruziziensis* for which tri-nucleotide repeat motif was the most frequent in these species [19,26,53]. This difference may also be caused by different parameters for detecting microsatellite and different genome composition of each species. The dominant di-nucleotide and tri-nucleotide repeat motif of *Dipteronia* was AG/CT and AAG/CTT which was consistent with studies using rubber tree [29,54]. The lowest frequent motif was CG/CG which was also rare in studies using the plants such as coffee, wheat, *Aspidistra saxicola*, peach, corn, soybean, rice and pear [47,55,56,57,58,59].

### 3.3. Polymorphic SSR Markers and Diversity Analyses of Dipteronia

In the present study, the success rate of working primer pairs was relatively high as compared to previous studies in other plants [34,45,50,60]. This high success rate suggested that the assembled sequences of *Dipteronia* in our study were reliable and effective. Interestingly, some of working primers generated fragments larger than the expected size. It was probably due to the result of an insertion among the amplified regions. From 374 working primers, we obtained 132 polymorphic genic SSR markers with the polymorphic proportion of 35.2%. The polymorphic rate of *Dipteronia* was lower than the previous studies using *Houttuynia cordata* (86%) and *Amorphophallus* (89.1%) [30,61]. Potentially, the lower polymorphic rate might be due to the limited sample sizes we collected and only two living species of this genus could be utilized. On the other hand, many of the primers that amplified discrete PCR bands from the two species did not show polymorphism in either species. After screening, we excluded 35 primers which showed polymorphism in one species but could not amplify unambiguous bands in another one, and then 97 primer pairs which showed high polymorphism were selected as genus-specific SSR markers to evaluate genetic diversity of *Dipteronia*. According to previous research, a PIC value greater than 0.5 generally indicates a highly polymorphic state [62]. In our study, 60 of these selected primers displayed PIC value greater than 0.5, indicating polymorphic SSR markers from this work were invaluable in marker-assisted *Dipteronia* genetic studies. The average alleles, in this study, were lower than genomic SSR markers from previous studies on *D. sinensis* (15.21) and *D. dyeriana* (12.3) [20,21]. This was partially due to the large sample sizes they used and the screening of genic SSRs with lower level of polymorphism in other research [44]. Genetic diversity analyses based on the selected polymorphic primers indicated that most of these SSR markers showed higher polymorphism in *D. sinensis* than that in *D. dyeriana*. It was probably because of the distribution range and natural populations of existing *D. dyeriana* are limited. According to the UPGMA analysis, we found that all populations were distinctly divided into two groups with bootstrap values of 100 (Figure 4). The result implied that there may exist high genetic variations or/and barriers between *D. sinensis* and *D. dyeriana*, which is consistent with previous studies based on cpSSR and AFLP [9,10]. In short, results from our study indicated the SSR markers from *Diperonia* transcriptome sequences are suitable and reliable. Therefore, our SSR markers derived from transcriptome sequences will be useful for detailed population genetic analyses of *Dipteronia* species.

## 4. Experimental Section

### 4.1. Plant Materials

Samples of *D. sinensis* and *D. dyeriana* for transcriptome sequencing were collected from the Botanic Garden in Xi’an, China and Kunming, China, in July 2014, respectively. Young leaves and fruits of an individual from both species were frozen in liquid nitrogen immediately and stored −80 °C prior to RNA extraction. We also collected 44 individuals from six natural populations of *D. sinensis* and four natural populations of *D. dyeriana*, respectively. All sampled individuals from each population were spaced at least 50 m apart. These materials were dried with silica gel for DNA extraction, PCR amplification, and SSR markers validation.

### 4.2. RNA Extracting, cDNA Library Construction and Illumina Paired-End Sequencing

Total RNA was extracted using the EASY spin microRNA Rapid extraction kit (Aidlab Biotech, Beijing, China) and stored at −80 °C until further use. The RNA quality was determined by gel electrophoresis and NanoDrop 2000 Spectrophotometer (Thermo Fisher Scientific, Wilmington, DE, USA). Purified RNA from leaves and fruits was pooled together with equal volume to construct cDNA libraries for transcriptome sequencing. In brief, mRNA was enriched using the NEBNext Poly(A) mRNA Magnetic Isolation Module (E7490, NEB, Ipswich, UK) from 5 μg of total RNA and sequencing libraries were prepared using NEBNext mRNA Library Prep Master Mix Set for Illumina (E6110, NEB,) and NEBNext Multiplex Oligos for Illumina (E7500, NEB,). Library insert sizes range from 100 to 200 bp. The insertion fragment sizes of prepared libraries were resolved on 1.8% agarose gel. Finally, size selected libraries were quantified using the Library Quantification Kit-Illumina GA Universal (KK4824, Kapa, Wilmington, DE, USA). The qualified libraries were amplified by bridge PCR to generate clustered template DNA fragments on the Illumina cbot. Ultimately, the clusters were sequenced on the Illumina Hiseq 2000 platform.

### 4.3. De Novo Assembly, Analysis of Sequences and Functional Annotation

Before assembly, the raw paired-end reads were filtered to obtain high-quality clean reads by removing adaptors, low-quality sequences (reads with unknown bases “N”), and reads with more than 20% low-quality bases (quality value ≤10). The high quality reads of *D. sinensis* and *D. dyeriana* were further separately assembled using Trinity with default parameters [63]. Afterwards, all the assembled unigene sets were pooled and assembled into non-redundant unigenes using the TIGR Gene Indices Clustering (TGICL) tools and CD-HIT program [64,65]. The parameters of TGICL were set at a similarity of 95% and an overlap length of 40 bp and the sequence identity cut-off for CD-HIT was set to 0.95. Finally, all non-redundant unigenes were searched against the NCBI’s non-redundant (Nr) protein database, Cluster of Orthologous Group (COG), Swiss-Prot and the Kyoto Encyclopedia of Genes and Genomes (KEGG) pathway database with an E-value threshold of 1E-5. Gene ontology (GO) annotation was implemented on Blast2GO [66,67,68] with a cut-off E-value of 1E-5 and then plotted with functional classification using WEGO [69].

### 4.4. Microsatellite Screening and Primer Design

Microsatellite screening was performed using the MISA program [70] with parameters for identifying SSR as six for di-, five for tri- and tetra-, four for penta- and hexa-nucleotide motifs, respectively. Mono-nucleotide repeats were excluded in our analyses. Primers were designed using Primer3 [71]. The criteria for designing primers were as follows: PCR product size range of 100 to 300 bp; primer length of 18–21 nucleotides; GC content of 40%–70% with 50% as optimum and annealing temperature between 50 and 70 °C with 55 °C as the optimum melting temperature.

### 4.5. DNA Isolation, PCR Amplification and SSR Validation

Plant DNA was isolated from dried leaves of 44 individuals from different populations using the Plant Genomic DNA Kit (TianGen Biotech Co., Ltd., Beijing, China). Gel electrophoresis was performed using 1% agarose gel to check DNA integrity. All SSR primers were tested for amplification using DNA from the two species. PCR amplifications were performed in a reaction volume of 10 μL with 5 μL 2 × Taq PCR Master Mix, 0.2 μM of each primer, 1 μL template DNA and 3.6 μL ddH_2_O. All amplifications were carried out in SimpliAmp™ Thermal Cycler (Applied Biosystems, Carlsbad, CA, USA) as follow: denaturation at 94 °C for 5 min, followed by 30 cycles of 94 °C for 50 s, at specific annealing temperature (Tm) for 30 s, 72 °C for 40 s and 72 °C for 5 min as final extension. PCR products were resolved on 10% denaturing polyacrylamide gels and stained using a silver-staining protocol. The size of the DNA bands was determined by a PBR322 marker ladder (TianGen Biotech) and the alleles were scored using Quantity One Software v. 4.6.2 (Bio-Rad Laboratories, Hercules, CA, USA).

### 4.6. Genetic Diversity Analysis and Microsatellite Scoring

Genetic analyses for polymorphic loci were performed using GenAlEx 6.501 [72] to calculate parameters such as the number of alleles, effective number of alleles, expected heterozygosity, observed heterozygosity and Shannon’s information index. The probabilities of deviation from Hardy-Weinberg equilibrium (HWE) for all microsatellite loci were determined using GenAlEx 6.501. CERVUS version 3.0.7 [73] was used to calculate the value of polymorphic information content (PIC) of each SSR primer. The UPGMA (unweighted pair-group method with arithmetic averaging) analysis based on Nei’s (1987) genetic distances among populations was performed using TFPGA software [74]. Bootstrapping analysis (10,000 re-samplings) was carried out using the same software [74] in which bootstrap values over 50 were considered significant and provided on the dendrogram. In order to test the intra-specific polymorphisms of SSR loci in each species, genetic diversity of two species was also separately analyzed using GenAlEx 6.501 and CERVUS version 3.0.7.

## 5. Conclusions

Our study is the first to implement transcriptome sequencing in an endangered Chinese endemic genus by using NGS technology. We have identified a large set of genic SSR markers for *Dipteronia* based on transcriptome analysis. In this study, a total of 99,358 non-redundant unigenes were obtained after assembly. A total of 52,789 unigenes sequences had at least one blast hit against the Nr, COG, Swiss-Prot, KEGG, or GO database. In addition, 12,377 microsatellite loci were detected from non-redundant unigenes and 4179 primer pairs were designed. We selected 435 primers to validate in multiple individuals of *Dipteronia* populations that resulted in 132 SSR polymorphic microsatellite markers. Our finding demonstrated that Illumina paired-end sequencing is a rapid and cost-effective way for identifying SSR markers in non-model organisms. The identified SSR markers are valuable for population genetic and evolutionary studies on *Dipteronia*.

## Supplementary Materials

Supplementary materials can be accessed at: http://www.mdpi.com/1420-3049/21/2/166/s1.
