# Supplementary material for: Transcriptome Sequencing and Development of Genic SSR Markers of an Endangered Chinese Endemic Genus Dipteronia Oliver (Aceraceae)
Source: Molecules. 2016 Feb 23;21(3):166. doi: 10.3390/molecules21030166 (PMC6272838; doi:10.3390/molecules21030166)
Supplement: Supplementary file 1 [file molecules-21-00166-s001.zip › molecules-112292-SI-layout/Figure S1,S2 Table S2,S3,S4.pdf]

Supplementary Materials: Transcriptome Sequencing and Development of Genic SSR Markers of an Endangered Chinese Endemic Genus *Dipteronia* Oliver (*Aceraceae*)

Tao Zhou, Zhong-Hu Li, Guo-Qing Bai, Li Feng, Chen Chen, Yue Wei, Yong-Xia Chang and Gui-Fang Zhao

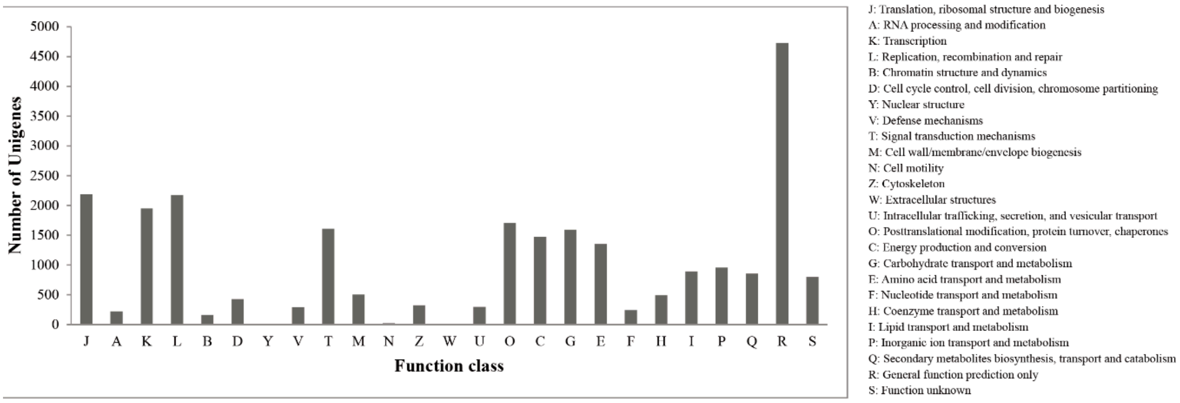

Figure S1. Clusters of orthologous groups (COG) classification of *Dipteronia* transcriptome. All the unigenes were aligned to COG database to predict and classify possible functions

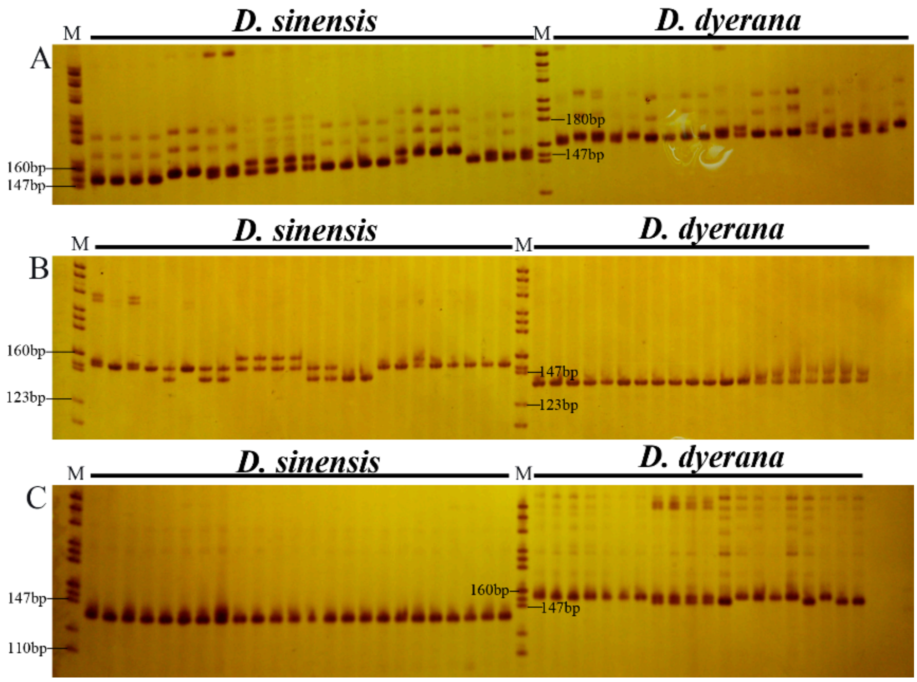

Figure S2. Polymorphism of primers among 44 individuals of *Dipteronia*. A. One of primers (DSSR2) which showed polymorphism in both *D. sinensis* and *D. dyeriana*. B. One of the primers (DSSR57) which showed a higher polymorphism in *D. sinensis*. C. One of the primers (DSSR86) which showed a higher polymorphism in *D. dyeriana*.

Table S2. Informativeness of genic-SSR loci following amplification from 44 individuals from different natural population of *Dipteronia.*

Locus	Na [1]	Ne [2]	H_O [3]	H_E [4]	I [5]	PIC [6]	HWE [7]
DSSR1	5	3.7556	0.159	0.742	1.4640	0.697	0.025347 *
DSSR2	6	4.3801	0.386	0.781	1.6175	0.738	0.500915
DSSR3	2	1.5708	0.023	0.368	0.5495	0.297	0.025347 *
DSSR4	5	2.8201	0.295	0.653	1.2123	0.579	0.709388
DSSR5	6	3.8489	0.091	0.749	1.5172	0.702	0.025347 *
DSSR6	11	8.6044	0.250	0.894	2.2365	0.872	0.025347 *
DSSR7	6	4.3022	0.045	0.776	1.5568	0.730	0.164476
DSSR8	13	6.5739	0.477	0.858	2.1365	0.831	0.124652
DSSR9	7	3.7886	0.455	0.745	1.5739	0.704	0.410859
DSSR10	11	4.3801	0.250	0.781	1.8329	0.749	0.57615
DSSR11	6	4.7393	0.205	0.798	1.6529	0.757	0.025347 *
DSSR12	5	4.7451	0.182	0.798	1.5841	0.756	0.654721
DSSR13	7	5.5393	0.159	0.829	1.8072	0.795	0.025347 *
DSSR14	5	3.2213	0.227	0.697	1.3007	0.638	0.025347 *
DSSR15	4	2.0607	0.227	0.521	0.9272	0.459	0.289756
DSSR16	6	4.0502	0.477	0.762	1.5508	0.715	0.427171
DSSR17	3	2.4694	0.045	0.602	0.9949	0.526	0.57615
DSSR18	7	2.9992	0.409	0.674	1.4037	0.632	0.504985
DSSR19	3	2.0817	0.045	0.526	0.7835	0.406	0.504985
DSSR20	3	2.1310	0.023	0.537	0.8165	0.422	0.350648
DSSR21	4	2.8618	0.045	0.658	1.1143	0.581	0.775097
DSSR22	3	2.3725	0.091	0.585	0.9348	0.486	0.504985
DSSR23	4	2.3438	0.227	0.580	1.0451	0.517	0.803785
DSSR24	6	3.3495	0.205	0.710	1.4080	0.659	0.025347 *
DSSR25	3	2.3048	0.273	0.573	0.9576	0.504	0.046012 *
DSSR26	7	3.2730	0.205	0.702	1.4447	0.650	0.775097
DSSR27	4	2.8325	0.205	0.654	1.1278	0.578	0.230139
DSSR28	6	3.1608	0.205	0.691	1.3498	0.634	0.230139
DSSR29	8	3.2953	0.364	0.705	1.5281	0.664	0.532104
DSSR30	4	2.9490	0.182	0.668	1.1698	0.597	0.442119
DSSR31	2	1.3653	0.136	0.271	0.4382	0.232	0.0455 *
DSSR32	3	2.3255	0.114	0.577	0.9156	0.475	0.350648
DSSR33	3	2.1310	0.068	0.537	0.8165	0.422	0.230139
DSSR34	4	2.8222	0.136	0.653	1.1301	0.580	0.0455 *
DSSR35	3	2.0817	0.045	0.526	0.7835	0.406	0.504985
DSSR36	6	1.9477	0.091	0.492	0.9898	0.454	0.2173
DSSR37	4	2.4884	0.091	0.605	1.0250	0.514	0.930916
DSSR38	3	2.3725	0.091	0.585	0.9348	0.486	0.230139
DSSR39	3	2.8119	0.250	0.652	1.0666	0.572	0.0455 *
DSSR40	3	2.3725	0.000	0.585	0.9348	0.486	
DSSR41	11	3.0730	0.409	0.682	1.6632	0.657	0.261464
DSSR42	5	3.2026	0.250	0.696	1.3331	0.645	0.230139
DSSR43	5	2.9467	0.114	0.668	1.2303	0.600	0.57615
DSSR44	4	1.7324	0.159	0.428	0.7935	0.388	1
DSSR45	3	1.2621	0.136	0.210	0.4305	0.197	0.025347 *
DSSR46	3	2.1802	0.091	0.548	0.8455	0.436	0.0455 *
DSSR47	4	3.0156	0.227	0.676	1.2117	0.610	0.350648
DSSR48	5	2.3283	0.250	0.577	0.9882	0.480	0.504985
DSSR49	3	2.2317	0.091	0.558	0.8718	0.450	0.775097

Table S2. *Cont.*

Locus	Na [1]	Ne [2]	H_O [3]	H_E [4]	I [5]	PIC [6]	HWE [7]
DSSR50	5	3.1100	0.023	0.686	1.2677	0.624	0.0455 *
DSSR51	4	2.5061	0.023	0.608	1.0177	0.519	0.046012 *
DSSR52	5	2.5357	0.068	0.613	1.1016	0.529	0.025347 *
DSSR53	4	2.6502	0.205	0.630	1.0988	0.548	0.504985
DSSR54	3	2.5676	0.136	0.618	1.0180	0.541	0.803785
DSSR55	4	2.9602	0.182	0.670	1.1983	0.602	0.527585
DSSR56	2	1.4824	0.227	0.329	0.5066	0.272	0.0455 *
DSSR57	3	2.2151	0.045	0.555	0.8811	0.456	0.709388
DSSR58	5	2.8681	0.091	0.659	1.2162	0.588	0.230139
DSSR59	9	3.6876	0.295	0.737	1.6617	0.703	0.261464
DSSR60	4	2.9157	0.136	0.665	1.1732	0.593	0.504985
DSSR61	7	3.3670	0.227	0.711	1.4636	0.662	0.504985
DSSR62	3	2.6575	0.114	0.631	1.0278	0.545	0.775097
DSSR63	3	2.1802	0.091	0.548	0.8455	0.436	0.0455 *
DSSR64	4	2.3960	0.136	0.589	0.9978	0.494	1
DSSR65	2	1.0465	0.045	0.045	0.1085	0.043	0.504985
DSSR66	2	2.0000	0.091	0.506	0.6931	0.375	0.0455 *
DSSR67	4	2.9762	0.341	0.672	1.2223	0.612	0.0455 *
DSSR68	6	3.0345	0.227	0.678	1.2781	0.614	0.230139
DSSR69	8	3.8073	0.114	0.746	1.6641	0.712	0.046012 *
DSSR70	4	2.0930	0.068	0.528	0.8500	0.427	0.775097
DSSR71	2	1.9197	0.159	0.485	0.6721	0.364	0.775097
DSSR72	8	3.5328	0.341	0.725	1.5733	0.683	0.261464
DSSR73	2	1.0950	0.000	0.088	0.1849	0.083	0.025347 *
DSSR74	3	2.5865	0.159	0.620	1.0076	0.532	0.504985
DSSR75	4	2.2291	0.205	0.558	0.8986	0.451	0.0455 *
DSSR76	3	2.0817	0.045	0.526	0.7835	0.406	0.504985
DSSR77	3	1.4556	0.182	0.317	0.5944	0.289	0.230139
DSSR78	3	2.8119	0.250	0.652	1.0666	0.572	0.0455 *
DSSR79	3	2.2776	0.136	0.567	0.8945	0.463	0.775097
DSSR80	8	3.5490	0.205	0.726	1.5777	0.684	0.261464
DSSR81	3	2.3960	0.091	0.589	0.9667	0.508	0.025347 *
DSSR82	4	2.3424	0.068	0.580	0.9699	0.481	0.775097
DSSR83	3	2.1032	0.023	0.531	0.8101	0.418	0.241489
DSSR84	3	2.0325	0.023	0.514	0.7442	0.390	0.775097
DSSR85	2	1.9959	0.045	0.505	0.6921	0.374	0.504985
DSSR86	5	4.3214	0.273	0.777	1.5275	0.731	0.025347 *
DSSR87	5	2.7191	0.341	0.639	1.2196	0.583	0.230139
DSSR88	4	2.4368	0.182	0.596	1.0157	0.519	0.484395
DSSR89	3	2.6233	0.136	0.626	1.0183	0.539	0.504985

Table S2. *Cont.*

Locus	Na [1]	Ne [2]	*Ho* [3]	*H*E [4]	I [5]	PIC [6]	HWE [7]
DSSR90	4	2.0930	0.091	0.528	0.8500	0.427	0.504985
DSSR91	5	3.4602	0.250	0.719	1.4133	0.672	0.350648
DSSR92	2	1.1200	0.068	0.108	0.2181	0.101	0.241489
DSSR93	3	2.4805	0.205	0.604	0.9989	0.529	0.803785
DSSR94	4	3.1051	0.023	0.686	1.2455	0.624	0.350648
DSSR95	3	2.4383	0.182	0.597	0.9833	0.518	0.654721
DSSR96	3	2.1032	0.023	0.531	0.8101	0.418	0.241489
DSSR97	4	2.4444	0.000	0.598	1.1105	0.547	0.025347 *

[1] number of alleles; [2] The number of effective number of alleles; [3] observed heterozygosity; [4] expected heterozygosity; [5] Shannon's Information Index; [6] Polymorphism information content; [7] Hardy–Weinberg equilibrium, the probabilities of deviation from Hardy–Weinberg equilibrium (HWE) are indicated by asterisks (* $p < 0.05$).

Table S3. Informativeness of genic-SSR loci following amplification from 24 individuals from different natural population of *D. sinensis.*

Locus	Na [1]	Ne [2]	*Ho* [3]	HE [4]	I [5]	PIC [6]	HWE [7]
DSSR1	2	1.6000	0.000	0.383	0.5623	0.305	0.046 *
DSSR2	3	2.2456	0.375	0.566	0.9407	0.493	0.505
DSSR3	2	1.6528	0.042	0.403	0.5841	0.317	0.351
DSSR4	3	1.6067	0.458	0.386	0.6885	0.344	0.505
DSSR5	3	1.7534	0.042	0.439	0.7645	0.388	0.046 *
DSSR6	7	5.1892	0.458	0.824	1.7492	0.780	0.558
DSSR7	2	1.9459	0.000	0.496	0.6792	0.368	0.046 *
DSSR8	6	3.3103	0.583	0.713	1.4440	0.661	0.285
DSSR9	2	1.4922	0.417	0.337	0.5117	0.275	0.230
DSSR10	8	4.9655	0.292	0.816	1.7686	0.771	0.172
DSSR11	6	4.2198	0.375	0.779	1.5933	0.730	0.172
DSSR12	3	2.9463	0.208	0.675	1.0897	0.587	0.046 *
DSSR13	4	2.9691	0.167	0.677	1.1996	0.600	0.505
DSSR14	5	4.0280	0.208	0.768	1.4422	0.708	0.775
DSSR15	4	1.4750	0.167	0.329	0.6417	0.298	0.775
DSSR16	5	2.9018	0.417	0.669	1.2423	0.593	0.261
DSSR17	1	1.0000	0.000	0.000	0.0000	0.000	
DSSR18	7	4.5896	0.750	0.799	1.6813	0.752	0.505
DSSR19	2	1.0868	0.083	0.082	0.1732	0.077	0.505
DSSR20	2	1.1327	0.042	0.120	0.2338	0.110	0.351
DSSR21	3	2.0832	0.083	0.531	0.7797	0.405	0.775
DSSR22	2	1.3846	0.167	0.284	0.4506	0.239	0.505
DSSR23	4	3.1736	0.375	0.699	1.2493	0.630	0.931
DSSR24	6	3.2727	0.375	0.709	1.3702	0.645	0.230
DSSR25	3	2.8800	0.500	0.667	1.0776	0.579	0.046
DSSR26	6	3.0078	0.375	0.682	1.3855	0.632	0.775
DSSR27	3	2.0317	0.375	0.519	0.8045	0.415	0.230
DSSR28	5	2.7106	0.375	0.645	1.2115	0.584	0.230
DSSR29	8	4.5176	0.667	0.795	1.7651	0.755	0.532
DSSR30	3	1.4049	0.167	0.294	0.5443	0.264	0.505
DSSR31	2	1.7041	0.250	0.422	0.6036	0.328	0.046 *
DSSR32	2	1.3318	0.208	0.254	0.4154	0.218	0.351
DSSR33	2	1.1327	0.125	0.120	0.2338	0.110	0.230
DSSR34	4	2.3415	0.250	0.585	0.9762	0.483	0.046 *

Table S3. *Cont.*

Locus	Na [1]	Ne [2]	*Ho* [3]	HE [4]	I [5]	PIC [6]	HWE [7]
DSSR35	2	1.0868	0.083	0.082	0.1732	0.077	0.505
DSSR36	6	3.3103	0.167	0.713	1.3696	0.649	0.217
DSSR37	3	1.5238	0.167	0.351	0.6160	0.307	0.931
DSSR38	2	1.3846	0.167	0.284	0.4506	0.239	0.230
DSSR39	2	1.9965	0.458	0.510	0.6923	0.375	0.046 *
DSSR40	2	1.3846	0.000	0.284	0.4506	0.239	
DSSR41	1	8.2286	0.750	0.897	2.2366	0.867	0.261
DSSR42	5	3.5015	0.458	0.730	1.3483	0.660	0.230
DSSR43	3	1.4527	0.083	0.318	0.5480	0.274	0.775
DSSR44	4	2.6483	0.292	0.636	1.0938	0.559	1.000
DSSR45	3	1.2915	0.250	0.230	0.4563	0.212	0.505
DSSR46	2	1.1803	0.167	0.156	0.2868	0.141	0.046
DSSR47	3	2.3802	0.417	0.592	0.9582	0.502	0.351
DSSR48	5	1.6387	0.458	0.398	0.8279	0.371	0.505
DSSR49	3	1.2872	0.167	0.228	0.4331	0.206	0.775
DSSR50	4	2.5888	0.042	0.627	1.0609	0.537	0.046
DSSR51	4	1.7323	0.042	0.432	0.7702	0.374	0.046
DSSR52	3	1.2374	0.125	0.196	0.4042	0.183	0.775
DSSR53	3	1.7428	0.375	0.435	0.7513	0.381	0.505
DSSR54	2	1.0868	0.000	0.082	0.1732	0.077	0.046
DSSR55	3	2.2677	0.333	0.571	0.9337	0.488	0.528
DSSR56	2	1.8824	0.417	0.479	0.6616	0.359	0.046
DSSR57	1	1.0000	0.000	0.000	0.0000	0.000	
DSSR58	4	2.0945	0.167	0.534	0.9666	0.471	0.230
DSSR59	9	5.4857	0.542	0.835	1.8811	0.794	0.261
DSSR60	3	2.1818	0.250	0.553	0.8877	0.460	0.505
DSSR61	6	3.2914	0.417	0.711	1.4202	0.659	0.505
DSSR62	2	1.7534	0.208	0.439	0.6211	0.337	0.775
DSSR63	2	1.1803	0.167	0.156	0.2868	0.141	0.046
DSSR64	3	1.4118	0.250	0.298	0.5661	0.272	1.000
DSSR65	2	1.0868	0.083	0.082	0.1732	0.077	0.505
DSSR66	2	1.1803	0.167	0.156	0.2868	0.141	0.046
DSSR67	4	3.0236	0.625	0.684	1.2043	0.607	0.046
DSSR68	5	2.4202	0.417	0.599	1.0799	0.524	0.230
DSSR69	8	5.8477	0.208	0.847	1.9003	0.808	0.046
DSSR70	4	1.8885	0.125	0.480	0.8492	0.416	0.775
DSSR71	2	1.6528	0.292	0.403	0.5841	0.317	0.775
DSSR72	7	3.8919	0.625	0.759	1.6213	0.715	0.261
DSSR73	1	1.0000	0.000	0.000	0.0000	0.000	
DSSR74	2	1.6528	0.292	0.403	0.5841	0.317	0.505
DSSR75	4	1.4826	0.375	0.332	0.6635	0.307	0.046
DSSR76	2	1.0868	0.083	0.082	0.1732	0.077	0.505
DSSR77	3	1.9862	0.333	0.507	0.8570	0.443	0.230
DSSR78	2	1.9965	0.458	0.510	0.6923	0.375	0.046
DSSR79	2	1.2800	0.250	0.223	0.3768	0.195	0.775
DSSR80	7	3.9588	0.375	0.763	1.6293	0.717	0.261
DSSR81	1	1.0000	0.000	0.000	0.0000	0.000	
DSSR82	3	1.3505	0.125	0.265	0.5150	0.244	0.775
DSSR83	1	1.0000	0.000	0.000	0.0000	0.000	
DSSR84	2	1.0425	0.042	0.042	0.1013	0.040	0.775

Table S3. *Cont.*

Locus	Na [1]	Ne [2]	*Ho* [3]	H_E [4]	I [5]	PIC [6]	HWE [7]
DSSR85	2	1.0868	0.083	0.082	0.1732	0.077	0.505
DSSR86	3	2.6667	0.500	0.638	1.0282	0.545	0.230
DSSR87	5	3.3488	0.625	0.716	1.3849	0.660	0.230
DSSR88	1	1.0000	0.000	0.000	0.0000	0.000	
DSSR89	2	1.7041	0.250	0.422	0.6036	0.328	0.505
DSSR90	4	1.8885	0.167	0.480	0.8492	0.416	0.505
DSSR91	4	3.6113	0.458	0.738	1.3279	0.672	0.351
DSSR92	1	1.0000	0.000	0.000	0.0000	0.000	
DSSR93	1	1.0000	0.000	0.000	0.0000	0.000	
DSSR94	3	2.5772	0.042	0.625	1.0203	0.542	0.351
DSSR95	1	1.0000	0.000	0.000	0.0000	0.000	
DSSR96	1	1.0000	0.000	0.000	0.0000	0.000	
DSSR97	3	2.5714	0.000	0.624	1.0114	0.535	

[1] number of alleles; [2] The number of effective number of alleles; [3] observed heterozygosity; [4] expected heterozygosity; [5] Shannon's Information Index, [6] Polymorphism information content; [7] Hardy–Weinberg equilibrium, the probabilities of deviation from Hardy–Weinberg equilibrium (HWE) are indicated by asterisks (* $p < 0.05$).

Table S4. Informativeness of genic-SSR loci following amplification from 20 individuals from different natural population of *D. dyerana*.

Locus	Na [1]	Ne [2]	*Ho* [3]	*HE* [4]	I [5]	PIC [6]	HWE [7]
DSSR1	4	2.9091	0.350	0.673	1.1452	0.587	0.025 *
DSSR2	3	2.1563	0.400	0.550	0.9139	0.477	0.501
DSSR3	2	1.4706	0.000	0.328	0.5004	0.269	0.025 *
DSSR4	2	1.2195	0.100	0.185	0.3251	0.164	0.709
DSSR5	3	2.2923	0.150	0.578	0.9045	0.469	0.025 *
DSSR6	4	3.5088	0.000	0.733	1.3055	0.661	0.025 *
DSSR7	4	2.5974	0.100	0.631	1.0941	0.544	0.164
DSSR8	7	3.3195	0.350	0.717	1.4517	0.652	0.125
DSSR9	5	3.2000	0.500	0.705	1.3327	0.644	0.411
DSSR10	3	1.2270	0.200	0.190	0.3944	0.177	0.576
DSSR11	2	1.9231	0.000	0.492	0.6730	0.365	0.025 *
DSSR12	2	1.8824	0.150	0.481	0.6616	0.359	0.655
DSSR13	3	2.5723	0.150	0.627	1.0205	0.543	0.025 *
DSSR14	2	1.7817	0.250	0.450	0.6306	0.342	0.025 *
DSSR15	3	2.6756	0.300	0.642	1.0322	0.548	0.290
DSSR16	5	4.7619	0.550	0.810	1.5828	0.756	0.427
DSSR17	2	1.9231	0.100	0.492	0.6730	0.365	0.576
DSSR18	1	1.0000	0.000	0.000	0.0000	0.000	
DSSR19	1	1.0000	0.000	0.000	0.0000	0.000	
DSSR20	1	1.0000	0.000	0.000	0.0000	0.000	
DSSR21	1	1.0000	0.000	0.000	0.0000	0.000	
DSSR22	1	1.0000	0.000	0.000	0.0000	0.000	
DSSR23	2	1.5355	0.050	0.358	0.5332	0.288	0.804
DSSR24	2	1.2195	0.000	0.185	0.3251	0.164	0.025 *
DSSR25	1	1.0000	0.000	0.000	0.0000	0.000	
DSSR26	1	1.0000	0.000	0.000	0.0000	0.000	
DSSR27	1	1.0000	0.000	0.000	0.0000	0.000	
DSSR28	1	1.0000	0.000	0.000	0.0000	0.000	
DSSR29	1	1.0000	0.000	0.000	0.0000	0.000	

Table S4. *Cont.*

Locus	Na [1]	Ne [2]	H_O [3]	H_E [4]	I [5]	PIC [6]	HWE [7]
DSSR30	3	2.2284	0.200	0.565	0.8909	0.461	0.442
DSSR31	1	1.0000	0.000	0.000	0.0000	0.000	
DSSR32	1	1.0000	0.000	0.000	0.0000	0.000	
DSSR33	1	1.0000	0.000	0.000	0.0000	0.000	
DSSR34	1	1.0000	0.000	0.000	0.0000	0.000	
DSSR35	1	1.0000	0.000	0.000	0.0000	0.000	
DSSR36	1	1.0000	0.000	0.000	0.0000	0.000	
DSSR37	1	1.0000	0.000	0.000	0.0000	0.000	
DSSR38	1	1.0000	0.000	0.000	0.0000	0.000	
DSSR39	1	1.0000	0.000	0.000	0.0000	0.000	
DSSR40	1	1.0000	0.000	0.000	0.0000	0.000	
DSSR41	1	1.0000	0.000	0.000	0.0000	0.000	
DSSR42	1	1.0000	0.000	0.000	0.0000	0.000	
DSSR43	2	1.5355	0.150	0.358	0.5332	0.288	0.576
DSSR44	1	1.0000	0.000	0.000	0.0000	0.000	
DSSR45	2	1.2195	0.000	0.185	0.3251	0.164	0.025 *
DSSR46	1	1.0000	0.000	0.000	0.0000	0.000	
DSSR47	1	1.0000	0.000	0.000	0.0000	0.000	
DSSR48	1	1.0000	0.000	0.000	0.0000	0.000	
DSSR49	1	1.0000	0.000	0.000	0.0000	0.000	
DSSR50	1	1.0000	0.000	0.000	0.0000	0.000	
DSSR51	1	1.0000	0.000	0.000	0.0000	0.000	
DSSR52	2	1.3423	0.000	0.262	0.4227	0.222	0.025 *
DSSR53	1	1.0000	0.000	0.000	0.0000	0.000	
DSSR54	2	1.9802	0.300	0.508	0.6881	0.372	0.804
DSSR55	1	1.0000	0.000	0.000	0.0000	0.000	
DSSR56	1	1.0000	0.000	0.000	0.0000	0.000	
DSSR57	2	1.3423	0.100	0.262	0.4227	0.222	0.709
DSSR58	1	1.0000	0.000	0.000	0.0000	0.000	
DSSR59	1	1.0000	0.000	0.000	0.0000	0.000	
DSSR60	1	1.0000	0.000	0.000	0.0000	0.000	
DSSR61	1	1.0000	0.000	0.000	0.0000	0.000	
DSSR62	1	1.0000	0.000	0.000	0.0000	0.000	
DSSR63	1	1.0000	0.000	0.000	0.0000	0.000	
DSSR64	1	1.0000	0.000	0.000	0.0000	0.000	
DSSR65	1	1.0000	0.000	0.000	0.0000	0.000	
DSSR66	1	1.0000	0.000	0.000	0.0000	0.000	
DSSR67	1	1.0000	0.000	0.000	0.0000	0.000	
DSSR68	1	1.0000	0.000	0.000	0.0000	0.000	
DSSR69	2	1.6000	0.000	0.385	0.5623	0.305	
DSSR70	1	1.0000	0.000	0.000	0.0000	0.000	
DSSR71	1	1.0000	0.000	0.000	0.0000	0.000	
DSSR72	1	1.0000	0.000	0.000	0.0000	0.000	
DSSR73	2	1.2195	0.000	0.185	0.3251	0.164	0.025 *
DSSR74	1	1.0000	0.000	0.000	0.0000	0.000	
DSSR75	1	1.0000	0.000	0.000	0.0000	0.000	
DSSR76	1	1.0000	0.000	0.000	0.0000	0.000	
DSSR77	1	1.0000	0.000	0.000	0.0000	0.000	
DSSR78	1	1.0000	0.000	0.000	0.0000	0.000	
DSSR79	1	1.0000	0.000	0.000	0.0000	0.000	

Table S4. *Cont.*

Locus	Na [1]	Ne [2]	H_O [3]	H_E [4]	I [5]	PIC [6]	HWE [7]
DSSR80	1	1.0000	0.000	0.000	0.0000	0.000	
DSSR81	2	1.7241	0.200	0.431	0.6109	0.332	0.025 *
DSSR82	1	1.0000	0.000	0.000	0.0000	0.000	
DSSR83	2	1.1611	0.050	0.142	0.2664	0.129	0.241
DSSR84	1	1.0000	0.000	0.000	0.0000	0.000	
DSSR85	1	1.0000	0.000	0.000	0.0000	0.000	
DSSR86	2	1.7241	0.000	0.431	0.6109	0.332	0.025 *
DSSR87	1	1.0000	0.000	0.000	0.0000	0.000	
DSSR88	3	1.8307	0.400	0.465	0.7187	0.371	0.484
DSSR89	1	1.0000	0.000	0.000	0.0000	0.000	
DSSR90	1	1.0000	0.000	0.000	0.0000	0.000	
DSSR91	1	1.0000	0.000	0.000	0.0000	0.000	
DSSR92	2	1.2800	0.150	0.224	0.3768	0.195	0.241
DSSR93	2	1.9560	0.450	0.501	0.6819	0.369	0.804
DSSR94	1	1.0000	0.000	0.000	0.0000	0.000	
DSSR95	2	1.8349	0.400	0.467	0.6474	0.351	0.655
DSSR96	2	1.1611	0.050	0.142	0.2664	0.129	0.241
DSSR97	2	1.7241	0.000	0.431	0.6109	0.332	0.025 *

[1] number of alleles; [2] The number of effective number of alleles; [3] observed heterozygosity; [4] expected heterozygosity; [5] Shannon's Information Index; [6] Polymorphism information content; [7] Hardy–Weinberg equilibrium, the probabilities of deviation from Hardy–Weinbergequilibrium (HWE) are indicated by asterisks (* $p < 0.05$).
